# Reduction of Growth and Reproduction of the Biotrophic Fungus *Blumeria graminis* in the Presence of a Necrotrophic Pathogen

**DOI:** 10.3389/fpls.2016.00742

**Published:** 2016-05-31

**Authors:** Elizabeth S. Orton, James K. M. Brown

**Affiliations:** Crop Genetics, John Innes CentreNorwich, UK

**Keywords:** powdery mildew, *Blumeria graminis*, *Zymoseptoria tritici*, co-infection, wheat, biotrophic pathogens, necrotrophic pathogens

## Abstract

Crops are attacked by many potential pathogens with differing life-history traits, which raises the question of whether or not the outcome of infection by one pathogen may be modulated by a change in the host environment brought on by infection by another pathogen. We investigated the host-mediated interaction between the biotroph *Blumeria graminis* f.sp. *tritici* (*Bgt*), the powdery mildew pathogen of wheat, and the necrotroph *Zymoseptoria tritici*, which has a long latent, endophytic phase following which it switches to a necrotrophic phase, resulting in the disease symptoms of Septoria tritici blotch. Both diseases are potentially severe in humid temperate climates and are controlled by fungicides and by growing wheat varieties with partial resistance. The compatible interaction between *Z. tritici* and the host reduced the number, size, and reproductive capacity of mildew colonies that a normally virulent *Bgt* isolate would produce but did not significantly alter the early development of *Bgt* on the leaf. The effect on virulent *Bgt* was elicited only by viable spores of *Z. tritici*. Notably, this effect was seen before the necrotic foliar symptoms induced by *Z. tritici* were visible, which implies there is a physiological interaction during the latent, endophytic period of *Z. tritici*, which either takes place directly between this fungus and *Bgt* or is mediated by the wheat leaf. Information on how different pathogens interact in host plants may allow plant breeders and others to improve the design of screening trials and selection of germplasm.

## Introduction

Plants are exposed to many different microbes including potential pathogens. Studies of disease on crop pathology tend to focus on individual diseases, but when environmental conditions are conducive to more than one parasite, plants must defend themselves against several species, often with different life histories. Understanding interactions between multiple pathogens on a host plant is essential for being able to control multiple diseases simultaneously in a crop. Disease control on arable crops generally requires the combined use of pesticides and resistant varieties (Torriani et al., 2015). While some groups of fungicides have broad-spectrum activity against several diseases,the scope for relying on them is diminishing because of the evolution of insensitivity in important pathogens (Lucas et al., 2015) and increased regulation of the marketing and use of agrochemicals (Hillocks, 2012; Jess et al., 2014). Regarding breeding for disease resistance, a cultivar has little value to farmers if it has good resistance to one disease but high susceptibility to another disease which is also important in the same environment. Efforts to breed crop varieties resistant to multiple pathogens will be assisted by information on how important pathogens interact with each other and the mechanisms behind their interaction. This will support the design of disease screening trials and the choice of parental germplasm.

The disease outcome of infection by one pathogen may be mediated by a change in host environment brought on by infection by another pathogen. Both induction and suppression of a plant’s defenses by prior contact with a pathogen has been reported. In the early mid 20th century several authors noted that cereals attacked by one pathogen predisposed them to attack by another (Bensaude, 1926; Chester, 1944; Yarwood, 1959; Brokenshire, 1974). More recently in tests in controlled environment conditions, it has been shown that biotrophic pathogens can suppress a plant’s resistance to both biotrophic and non-biotrophic pathogens; *Albugo candida* suppresses innate immunity allowing infection by avirulent *Hyaloperonospora arabidopsidis* (Cooper et al., 2008) while *Pseudomonas syringae* pv. t*omato* (*Pst*) induces salicylic acid-mediated defenses and suppresses the jasmonic acid-mediated defense pathway normally induced in plants infected with *Alternaria brassicicola* (Spoel et al., 2007).

A reduction in disease severity has also been noted in field conditions. The non-biotrophic fungus *Parastagonospora nodorum* reduced the disease severity of powdery mildew (*Blumeria graminis*) although the presence of *B. graminis* increased the accumulated disease severity caused by *P. nodurum* (Weber et al., 1994). In a glasshouse trial, the non-biotrophic fungal pathogen *Zymoseptoria tritici* reduced the incidence of *Puccinia striiformis*, thought to be due to competition between the two pathogens (Madariaga and Scharen, 1986). Under controlled conditions, Lyngkjær and Carver (1999) found that an initial successful infection by a virulent isolate of *B. graminis* can render cells accessible to future attacks by other, normally avirulent isolates of this fungus but equally, inaccessibility to future infection could be induced if the initial attack failed. Aghnoum and Niks (2012), investigating interactions between virulent *P. hordei* and *B. graminis* f.sp. *hordei* isolates on barley, found that pre-inoculation with the rust isolate induced increased resistance to both isolates of *B. graminis* by preventing haustorium formation.

These previous studies demonstrate that the interactions between pathogens are not yet predictable and that there are many underlying factors in the outcome of infection. The aim of this research was to investigate how infection of wheat with *Z. tritici* affects the wheat plant’s response to *B. graminis* f.sp. *tritici* (*Bgt*). *Z. tritici* causes the disease Septoria tritici blotch, currently the most common foliar disease of wheat in temperate areas in much of the world (Fones and Gurr, 2015). Efficacy of fungicides is declining for control of this disease as the population develops insensitivity to triazole and strobulurin fungicides (Fraaije et al., 2005, 2007) while there has been a recent report of insensitivity to succinate dehydrogenase fungicides (Teagasc, 2015). Currently a combination of several fungicides still gives good control of Septoria on moderately resistant wheat varieties (Torriani et al., 2015). Breeding is improving the level of resistance to Septoria, but not to the extent that fungicides can be dispensed with (Brown, 2015). There is also widespread insensitivity of wheat powdery mildew to broad-spectrum fungicides but it is generally well-controlled by a combination of specific anti-mildew fungicides (AHDB, 2016) and partial resistance, which is generally durable (Brown, 2015).

The two pathogens have very different modes of infection. *Z. tritici* enters the leaf through the stomata (Kema et al., 1996) and no differences have been seen in the penetration ability of avirulent and virulent *Z. tritici* spores (Shetty et al., 2003). This is followed by a long latent, apparently endophytic period (Orton et al., 2011; Sánchez-Vallet et al., 2015) in which the fungus remains within the substomatal cavity for at least 7 days after inoculation (dai) before symptoms appear on the leaf (reviewed by Steinberg, 2015). During this latent period there is no detectable increase in fungal biomass (Keon et al., 2007; Shetty et al., 2007). A compatible interaction is initiated with the onset of host cell collapse and growth of the fungus in the mesophyll layer between 10 and 14 dai, after which pycnidia are formed, emerging through the stoma after at least 14 days (Kema et al., 1996; Shetty et al., 2003; Keon et al., 2007). In an incompatible interaction, no increase in fungal biomass is seen (Shetty et al., 2007) and there is no evidence for macro- or microscopic symptoms indicative of a hypersensitive reaction (Hammond-Kosack and Rudd, 2008). *Bgt*, by contrast, grows on the epidermis, infecting cells from appressoria formed approximately 12 h after inoculation (hai). Haustoria are formed from 24 hai onwards within host cells, enabling the fungus to feed (Zhang et al., 2005). Except for the haustoria, which occupy the epidermal cells, the fungus grows on the surface of the leaf throughout its lifecycle. Asexual conidiophores are produced on the surface of the leaf from 5 to 10 dai. The hypersensitive response which forms during an incompatible interaction is a critical aspect of resistance to mildew (Boyd et al., 1995).

As these two pathogens cause widespread foliar diseases of wheat which often occur in the same mild, humid environments but have strongly contrasting lifestyles, their interaction is potentially of profound importance for attempts to control a broad spectrum of diseases by a combination of resistance breeding and chemical applications. We investigated the effect of a compatible interaction between the necrotroph *Z. tritici* and the wheat host on inhibiting the growth and development of a virulent isolate of the obligately biotrophic *Bgt*. This effect occurred several days before necrotic Septoria lesions formed, implying that signals which form during the early stages of the wheat-*Z. tritici* interaction, not the later formation of necrotic lesions, are responsible for inhibiting *Bgt*. Conversely, we tested if the maintenance of green leaf tissue during the incompatible interaction of Septoria-resistant wheat with an avirulent *Z. tritici* isolate (Kema et al., 1996; Shetty et al., 2003) promoted greater susceptibility of the leaf to an avirulent *Bgt* isolate, possibly by overriding the hypersensitive response to avirulent *Bgt.*

## Materials and Methods

### Plant and Fungal Material

The wheat varieties Longbow and Flame were used throughout these experiments. Flame has the mildew resistance gene *Pm4b* and Longbow carries *Pm2.* The *Bgt* isolate JIW11 is avirulent to both these genes while JIW48 is virulent to both of them. The mildew-susceptible cultivar Cerco was used as a control. Longbow, which has the Septoria resistance gene *Stb15*, is susceptible to *Z. tritici* isolate IPO323 while Flame, which has *Stb6*, is resistant.

Wheat plants were grown in a growth room with temperatures set to 18°C for 16 h light and 12°C in the dark for 8 h. *Z. tritici* isolate IPO323 (Kema and van Silfhout, 1997) was grown on YPD+ agar. For plant inoculation, the second leaves of 14 days old seedlings were attached adaxial side up to Perspex sheets using double-sided tape (Keon et al., 2007). The leaves were inoculated evenly with a fungal spore solution at a density of 10^7^ spores per mL of water using a swab stick with a cotton sterile tip (Fisher Scientific, Loughborough, Leicestershire, UK; Keon et al., 2007). Plants inoculated with *Z. tritici* were placed in trays with plastic lids and under black plastic sheeting, to achieve dark conditions with high relative humidity for 24 h. Control leaves were mock inoculated with water only. A method using detached leaves (Arraiano et al., 2001) was modified to test infection of wheat by two pathogens. The bottom of rectangular clear polystyrene boxes were filled with 50 mL 1% water agar with 100 mg L^-1^ benzimidazole. After 24 h in the dark the inoculated leaves were placed into the boxes and the cut ends of the leaves were covered with a layer of benzimidazole agar. *Bgt* isolates JIW11 and JIW48 were maintained on the wheat cultivar Cerco and were inoculated by blowing fresh spores into settling towers placed over the plant material (Boyd et al., 1994). For all experiments control boxes to check for mildew colony formation and *Z. tritici* sporulation were set up. Boxes were placed in a controlled environment cabinet at 14°C 9 h light supplemented with UV light and 15 h dark at 9°C.

### Effect of Decreasing Concentrations of *Z. tritici*

To observe if there is a dosage effect of *Z. tritici* spores on the number of colonies formed by virulent *Bgt*, leaves of Longbow were inoculated with decreasing concentrations of the *Z. tritici* IPO323 and placed into detached leaf boxes before inoculation with *Bgt*. The dilution series started at 10^7^ spores per mL and inoculum was successively diluted 2.5-fold down to 100 spores per mL (Keon et al., 2007). Each detached leaf box contained a leaf of Cerco and 10 leaves of Longbow encompassing the whole dilution series. The boxes were inoculated with the virulent *Bgt* isolate JIW48 under settling towers, 4 days after inoculation with *Z. tritici*. Colony formation was assessed by counting visible colonies under a 2× magnifying lens after 8–10 days. The experiment was done in three replicates.

### Requirement for Living *Z. tritici* Spores

To produce non-viable spores, a spore suspension of *Z. tritici* (IPO323) was autoclaved at 121°C at 15 psi for 15 min. The spore suspension was then inoculated onto leaves of Flame and Longbow and the leaves placed into detached leaf boxes, along with mock-inoculated leaves, before being inoculated with *Bgt* at 1 day after and 10 days after inoculation with the autoclaved *Z. tritici*. Two leaves of each treatment were included in each box and the experiment was carried out a total of three times.

### Early Development of *B. graminis*

**FIGURE 1:**
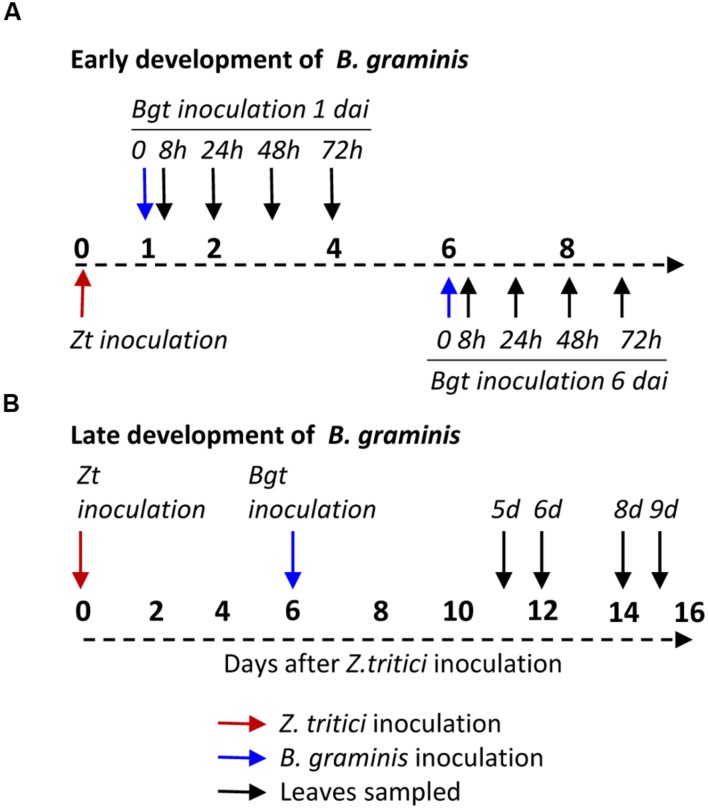
**Experimental design to investigate early and late development of *Blumeria graminis* f.sp. *tritici* on wheat leaves after inoculation with *Zymoseptoria tritici*.** **(A)** Inoculation and data collection points to study the early development of *Bgt* on wheat leaves after inoculation with *Z. tritici* at day 0. **(B)** Inoculation and data collection points to study the late development of *Bgt* on wheat leaves after inoculation with a *Z. tritici* at day 0. In this figure, dai refers to days after inoculation with *Z. tritici*. h and d refer to hours or days at which leaves were sampled following inoculation with *Bgt*.

To investigate the development of *Bgt* spores at the early stages of development on leaves preinoculated with *Z. tritici*, Longbow and Flame leaves inoculated with IPO323 or mock-inoculated were placed in detached leaf boxes along with leaves of Cerco, which were not inoculated with *Z. tritici*. Leaves were inoculated with the mildew isolate JIW48, 1 or 6 days after *Z. tritici* inoculation. Each of three replicate experiments consisted of three independent replicate detached leaf boxes, with each box containing one leaf of each variety given each treatment. Leaves were destructively sampled at 8 h, 24 or 32, 48, and 72 h after infection (hai) with *Bgt* (**Figure 1A**). After the first replicate was assessed at 24 h, it was decided that 32 h would be a better timepoint to sample at as more development of the *Bgt* germlings had taken place. In terms of mildew development, the 24 and 32 h time points are fairly close and in the statistical analysis each replicate was treated as a block effect. The sampled leaves were placed onto filter paper soaked in 3:1 ethanol: acetic acid until they had cleared and were stored in lactoglycerol (1:1:1 solution of lactic acid, glycerol and water) until assessment by microscopy. To visualize fungal spores, the leaves were placed on a glass slide and Aniline Blue 0.1% (made up in lactoglycerol) was pipetted onto them. On each leaf, 30 *Bgt* spores were assessed for growth and development at the following stages; no germination, primary germ tube, appressorial germ tube, appressorium, balloon haustorium, digitate haustorium, or elongating secondary hyphae (ESH). Only spores that were isolated, undamaged and not infecting the same cell as another spores were assessed. Observations were made using a Nikon Microphot-SA (2) general light microscope. Haustoria were visualized under differential interference contrast (DIC) microscopy where necessary.

Separate statistical analyses were conducted on the data at each timepoint. The categories of *Bgt* development were formed into groups. At 8 h, the number of spores that had germinated with either a primary germ tube or an appressorium was studied as a proportion of the total spores counted. The 24/32 h timepoints were combined and the proportion of spores that had infected the host, having formed at least a haustorium, was analyzed as a proportion of all germinated spores. At 48 and 72 h, the categories were grouped to analyze spores that had developed ESH as a proportion of the total number of infecting spores. For each timepoint, a logistic regression model was fitted with a binomial distribution, the model being Replicate + Day^∗^Treatment, where the crossing operator (^∗^) indicates that both the main effects and the interaction of the factors were estimated. Treatments were Longbow inoculated with IPO323, Flame inoculated with IPO323 or mock-inoculated Longbow. Standard errors were calculated on a logit scale and back-transformed predicted means were calculated for the purposes of presentation.

### Later Development of *B. graminis*

To investigate the effect of pre-inoculation with virulent *Z. tritici* on the later stages of development of *Bgt* colonies, leaves of Longbow were inoculated either with *Z. tritici* isolate IPO323 or mock inoculated, in the same way as the early stage development inoculations. Leaves were subsequently inoculated with virulent *Bgt* isolate JIW48, 6 dai with *Z. tritici.* Each box contained two leaves of each treatment. Two leaves of each treatment were sampled at 5, 6, 8, and 9 days after infection (dai) with *Bgt* (**Figure 1B**). The experiment was done in three replicates.

All mildew colonies on the leaves were measured using a graticule at 100× magnification. The area of the colony was calculated by assessing the area as that of an ellipse π*(ab)* where *a* and *b* are half the ellipse’s major and minor axes, respectively. The number of conidiophores was assessed on a scale of 0–4: (0 = zero, 1 = < 5, 2 = 5–10, 3 = 11–30, and 4 = 30+ conidiophores per colony). The data were analyzed using linear modeling using the model Treatment^∗^Day, where the Treatment factor indicated whether the leaves were inoculated with IPO323 or mock-inoculated. Colony sizes were transformed to square roots for statistical analysis. This normalized the variance and made it independent of fitted values. In addition, this procedure reflects the constant radial growth rate of mildew colonies. Least significant differences of predicted means were calculated at the 5% level.

### DNA Quantification to Determine *Bgt* Biomass

To determine the relative biomass of *Bgt*, DNA of infected wheat leaves was extracted and the amount of mildew DNA quantified using a Taqman probe assay (Fraaije et al., 2006). To check that isolate JIW48 contained the same cytochrome *b* gene fragment that is amplified by the primers, the fragment was cloned and sequenced. DNA was extracted from leaves with visible sporulating mildew colonies of isolate JIW48 using a Qiagen DNeasy kit (Qiagen, Valencia, CA, USA). A 136bp fragment of the cytochrome *b* gene was amplified with primers PMR1 (5′-TTACTGCATTCCTGGGTTATGTATTG-3′) and PMS1 (5′-CAGAGAAACCTCCTCAAAGGAACT -3′; Fraaije et al., 2006). The fragments amplified were cloned into pGEM-T easy vector (Promega, Madison, WI, USA) following the manufacturer’s protocol. The vector was transformed into OneShot TOP10/P3 competent cells (Invitrogen, Carlsbad, CA, USA) following the manufacturer’s protocol using a heat shock transformation procedure. Blue/white colony selection was used to select for transformed cells, which were purified using Qiagen MinElute plasmid purification kit (Qiagen, Valencia, CA, USA) and sent for sequencing at TGAC, Norwich, UK. Sequences were aligned using WebPrank using the default settings^1^ to four known *Bgt* cytochrome *b* sequences from different isolates available on the NCBI database^2^: Fel08 (AF343442.1), Fel12 (AF343441.1), JAS501 (AJ293567.1), and W26 (AJ293566.1).

Longbow leaves were inoculated with *Bgt* either 1 or 6 days after inoculation with *Z. tritici* and samples collected at 5 and 10 days after *Bgt* inoculation. Total DNA was quantified on a Picodrop spectrophotometer (Picodrop Ltd, Hinxton, UK) and diluted so each sample contained 10 ng/μL. The reaction mixture for qPCR contained 0.5 μM forward primer (PMR1), 0.3 μM reverse primer (PMS1), 0.1 μM of 5′-CY5/3′-BHQ2-labeled probe (5′-CTTGTCCTATTCATGGTATAGCGCTCATTAGG-3′) and 20 ng of DNA sample and 10 μL iQ supermix (Bio-Rad, Hemel Hempstead, Herts, UK) to a volume of 20 μL. A standard curve was produced by plotting known amounts of DNA against Cq values. The reaction cycle was: 2 min at 50°C, 2 min 95°C followed by 50 cycles of 15 s at 95°C and 1 min at 60°C. The increase in fluorescence from the probe was recorded at 60°C during every cycle.

A logistic regression model was fitted: Interval^∗^daiBg^∗^Trt where the ^∗^ operator indicates that both the main effects and the interaction of the factors were estimated. Interval indicates the amount of time between inoculation with *Z. tritici* and inoculation with *Bgt* (either 1 or 6 days). DaiBg is the time that the samples were taken, either 5 or 10 days after *Bgt* inoculation. Trt is the treatment of *Z. tritici* or mock inoculation. Standard errors were calculated using least significant differences of predicted means on a log10 scale and back-transformed for the purposes of presentation.

## Results

### Suppression of Mildew by Preinoculation with *Z. tritici*

**FIGURE 2:**
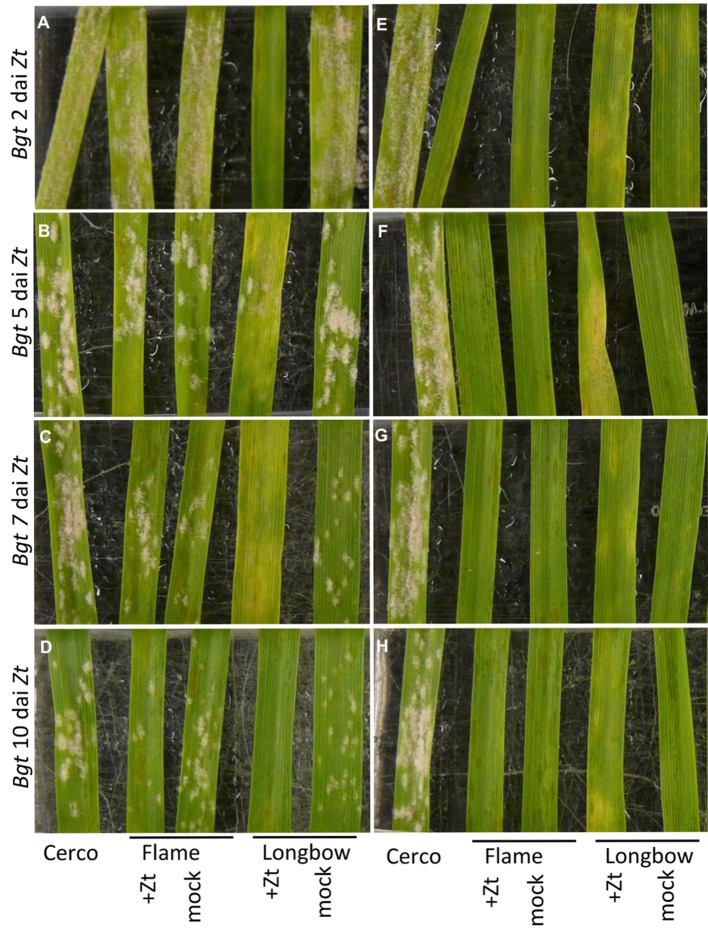
**Leaves of wheat varieties Flame and Longbow inoculated first with *Z. tritici* isolate IPO323 then with either avirulent or virulent isolates of *B. graminis* f.sp. *tritici* (*Bgt*).** Order of leaves in each photograph (L to R): Cerco, Flame + IPO323, mock-inoculated Flame, Longbow + IPO323, Longbow mock. **(A–D)** Inoculated with virulent *Bgt* isolate JIW48. **(E–H)** Inoculated with avirulent *Bgt* isolate JIW11. Leaves were inoculated with *Bgt* 2 days after inoculation (dai) with *Z. tritici* **(A,E)**, 5 dai **(B,F)**, 7 dai **(C,G)** or 10 dai **(D,H)**. Photographed 20 days after inoculation with *Z. tritici*.

When Longbow was inoculated first with *Z. tritici* isolate IPO323 and subsequently with the virulent *Bgt* isolate JIW48, fewer or no mildew colonies were visible on the leaf than on the mock-inoculated controls. This result was consistent, regardless of whether the *Bgt* inoculation was carried out 2, 5, 7 or 10 dai with *Z. tritici*. When Flame was pre-inoculated with IPO323, and subsequently inoculated with JIW48, the number of mildew colonies on pre-inoculated leaves across all replicates was similar to that on mock-inoculated leaves (**Figures 2A–D**). When Flame and Longbow were pre-inoculated with *Z. tritici* and subsequently inoculated with an avirulent *Bgt* isolate, JIW11, no colonies of mildew formed (**Figures 2E–H**). Some chlorotic flecking was seen on leaves of Longbow inoculated first with *Z. tritici*, consistent with an incompatible response to avirulent *B. graminis* (**Figure 2F**).

**FIGURE 3:**
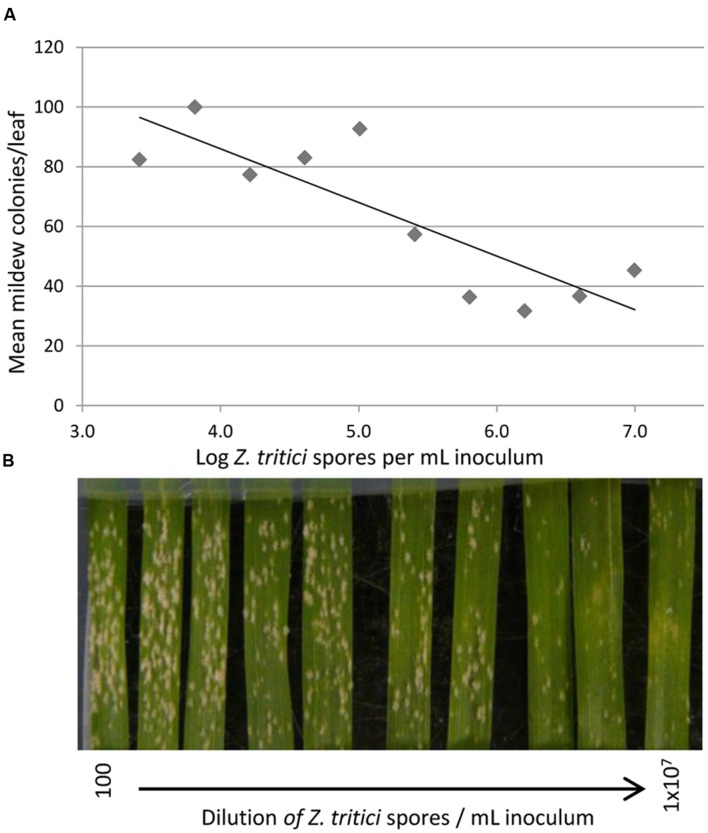
**The effect of different concentrations of virulent *Z. tritici* inoculum on the number of visible colonies of virulent *B. graminis* f.sp. *tritici.*** Leaves of Longbow were inoculated with decreasing concentrations of *Z. tritici* spores, from 1 × 10^7^/mL down to 100/mL. **(A)** A reduction of 18 mildew colonies per leaf was seen for every 10-fold increase in *Z. tritici* spores: the regression equation of the number of *Bgt* colonies **(B)** on the log_10_-concentration of *Z. tritici* (Z) was *B* = 158-18Z, *R*^2^ = 0.71. (*P* = 0.002 for linear regression). **(B)** Effect of increasing *Z. tritici* concentration on mildew colony formation. Photographed 14 days after inoculation with *Z. tritici*; 10 days after inoculation with *Bgt.*

When Longbow was inoculated with a dilution series of the virulent *Z. tritici* isolate, higher concentrations of *Z. tritici* spores hindered the formation of colonies formed by a virulent *Bgt* isolate more strongly (**Figure 3**, *P* = 0.002). The regression equation of the number of *Bgt* colonies (B) on the log_10_-concentration of *Z. tritici* (Z) was *B* = 158-18Z, implying that for every 10-fold reduction in *Z. tritici* spores, there were 18 more mildew colonies per leaf on average.

**FIGURE 4:**
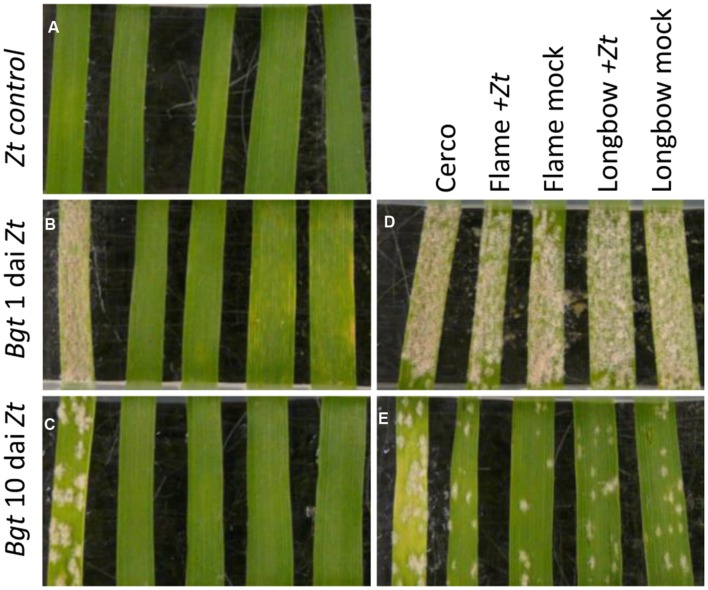
**The effect of non-viable *Z. tritici* spores on mildew colony development.** Order of leaves in each photograph, from left: Cerco, Flame IPO323, Flame mock, Longbow IPO323, Longbow mock. **(A)** Inoculated only with non-viable *Z. tritici* spores. **(B)** and **(C)** Inoculated with non-viable *Z. tritici* then with avirulent *B. graminis* f.sp. *tritici* (*Bgt*) isolate JIW11. **(D)** and **(E)** Inoculated with non-viable *Z. tritici* virulent *Bgt* isolate JIW48. **(B)** and **(D)** Inoculated with *Bgt* 1 day after *Z. tritici* inoculation. **(C)** and **(E)** inoculated with *Bgt* 10 days after *Z. tritici* inoculation. Photographed 21 days after inoculation with *Z. tritici.*

When Flame and Longbow were inoculated with a suspension of non-viable spores of IPO323, the appearance of mildew colonies on the leaves was similar to that on the mock inoculated leaves (**Figure 4**). The leaves were either inoculated after 1 or 10 dai with the non-viable *Z. tritici* spores; at 10 days, less mildew developed on all the leaves inoculated with the virulent mildew including the control cultivar Cerco.

### Early Development of *B. graminis*

**FIGURE 5:**
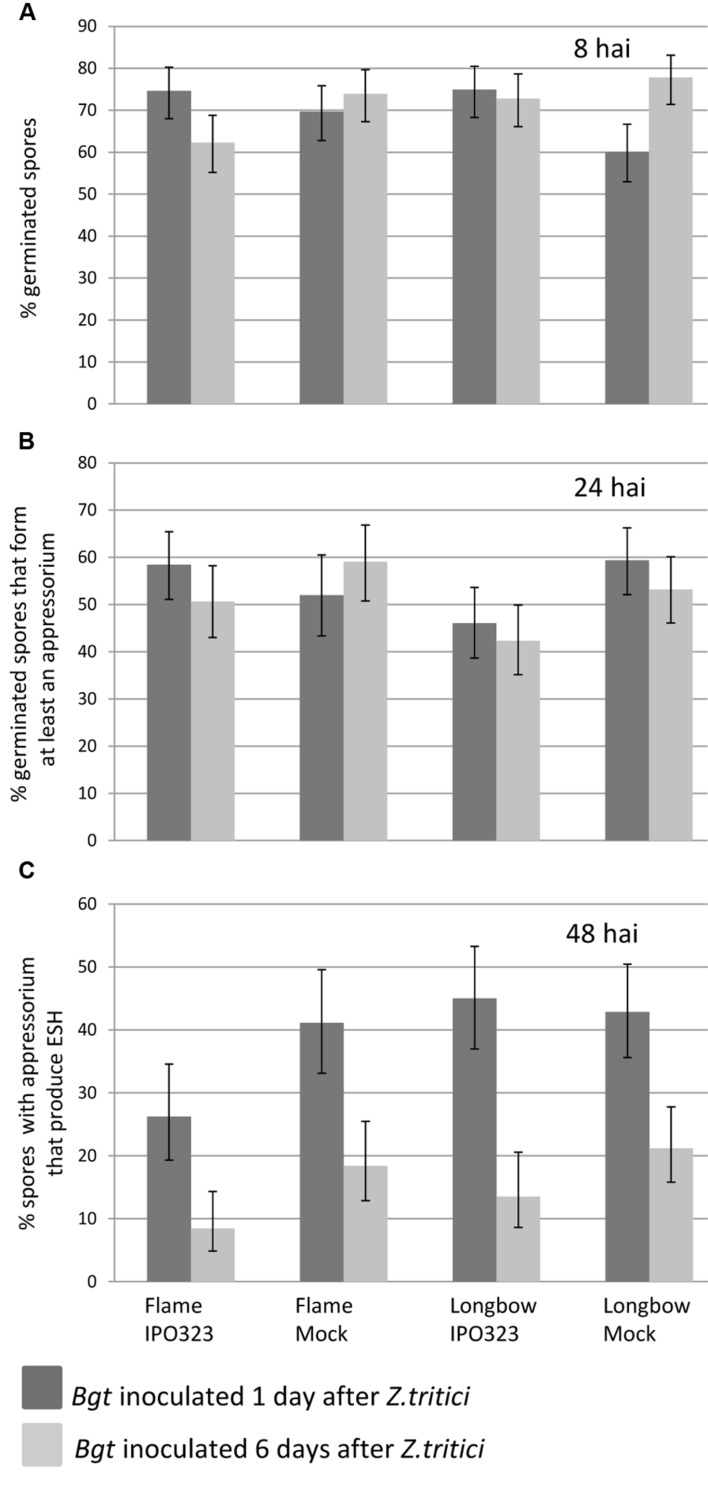
**The effect of inoculation with virulent *Z. tritici* on the early development of *B. graminis* f.sp. *tritici* (*Bgt*) on wheat leaves.** **(A)** Percentage of germinated spores 8 h after inoculation (hai) with *Bgt*. **(B)** Percentage of germinated spores that form at least an appressorium up to 24/32 hai with *Bgt*. **(C)** Percentge of spores which form an appressorium that produce elongated secondary hyphae (ESH) at 48 hai with *Bgt*. Error bars indicate ±1 standard error of predicted mean.

To assess the progress of *Bgt* spore development at the early stages of infection on leaves pre-inoculated with *Z. tritici*, leaves were sampled 8, 24/32, 48, and 72 hai after inoculation with *Bgt*, the leaves having been inoculated with *Z. tritici* either 1 or 6 days previously. At 8 hai, *Bgt* germination rates (spores scored as having at least a PGT) on leaves of all varieties and treatments ranged from 60 to 74.9% for leaves inoculated with *Bgt* 1 dai with *Z. tritici* and 62.3% to 77.8% for leaves inoculated 6 dai with *Z. tritici* (**Figure 5A**). There were no significant differences in the proportion of spores that had germinated between the treatments: Longbow with compatible *Z. tritici* IPO323, Longbow and Flame with a mock inoculation and Flame with incompatible IPO323 (*P* = 0.8; **Supplementary Table S1**). There were also no significant differences between the leaves inoculated with *Bgt* at 1 or 6 dai with *Z. tritici* (*P* = 0.68).

At 24/32 hai, a proportion of *Bgt* spores had attempted infection or succeeded in infecting the host, producing appressoria and occasionally haustoria and hyphae. The percentage of germinated spores that had formed at least an appressorium ranged from 46.0 to 59.4% on leaves inoculated with *Bgt* 1 dai with *Z. tritici* and from 42.3 to 59.0% on leaves inoculated with *Bgt* 6 dai with *Z. tritici* (**Figure 5B**). No significant differences in the proportion of germinated spores which had infected the plant were seen either between treatments (*P* = 0.3) or between days (*P* = 0.6).

At 48 hai, the percentage of *Bgt* spores which had formed an appressorium that had then gone on to form ESH ranged from 26.2 to 42.9% at 1 dai with *Z. tritici* and 8.4 to 21.1% at 6 dai with *Z. tritici* (**Figure 5C**). There were no significant differences between treatments (*P* = 0.2), but there was a significant effect of day (*P* < 0.001): consistently fewer *Bgt* spores produced ESH when infected with *Bgt* 6 dai with *Z. tritici* than 1 dai with *Z. tritici.* The data at 72 hai (not shown) were very similar to 48 hai.

### Later Development of *B. graminis*

**FIGURE 6:**
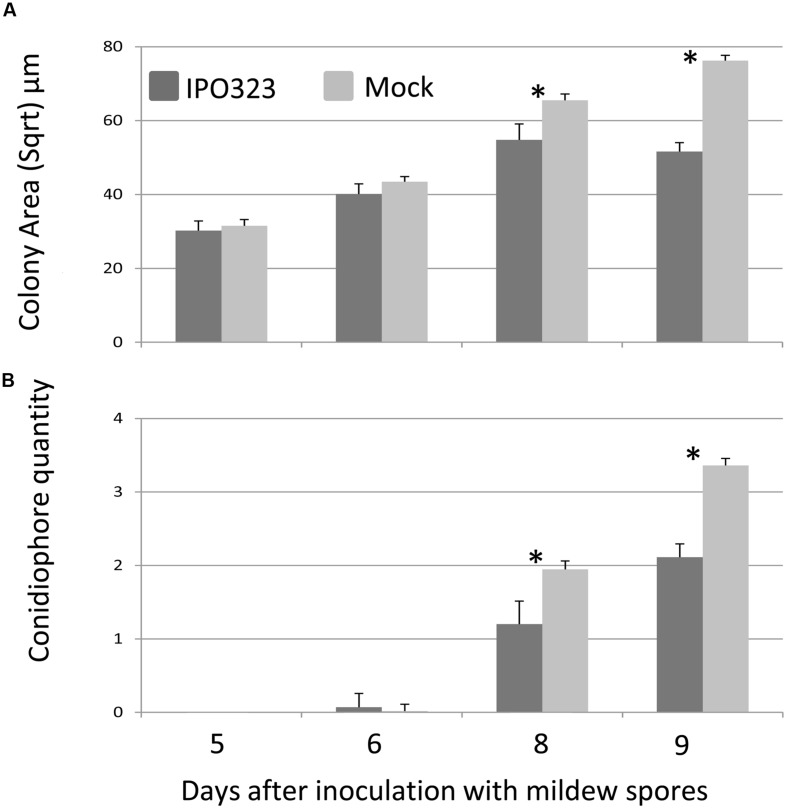
**Effect of pre-inoculation with virulent *Z. tritici* on the late stages of development of *B. graminis* f.sp. *tritici* (*Bgt*).** Leaves of Longbow were sampled 5–9 days after inoculation with *Bgt*, after either pre-inoculation with *Z. tritici* isolate IPO323 or mock-inoculation. **(A)** Mean square root of colony area (μm) where the square root of the area is proportional to the length of the axis of the ellipse formed by the colony. **(B)** Mean number of conidiophores per colony (0–4 scale). Error bars are ±1 SE of predicted means. ^∗^*P* < 0.05 (Fisher’s protected least significant difference).

To assess the effect of pre-inoculation with *Z. tritici* on the later stages of mildew colony development on the variety Longbow, the area of each mildew colony formed was measured at 5, 6, 8, and 9 days after inoculation with *Bgt.* At 5 and 6 days, pre-inoculation with virulent *Z. tritici* had no effect on the area of the colonies produced. By 8 days, the difference between the two treatments was significant and by 9 days, the gap between the two treatments was wider, with a difference of 605 μm^2^ between them (**Figure 6**). At 8 and 9 days after inoculation with *Bgt*, the number of conidiophores produced by the colonies on mock inoculated leaves was significantly greater than on the pre-inoculated leaves (**Figure 6**). At 9 days, the mock inoculated leaves produced between 10 and 30 conidiophores per colony, while the pre-inoculated leaves only produced up to 10 conidiophores per colony.

### DNA Quantification to Determine *Bgt* Biomass

**FIGURE 7:**
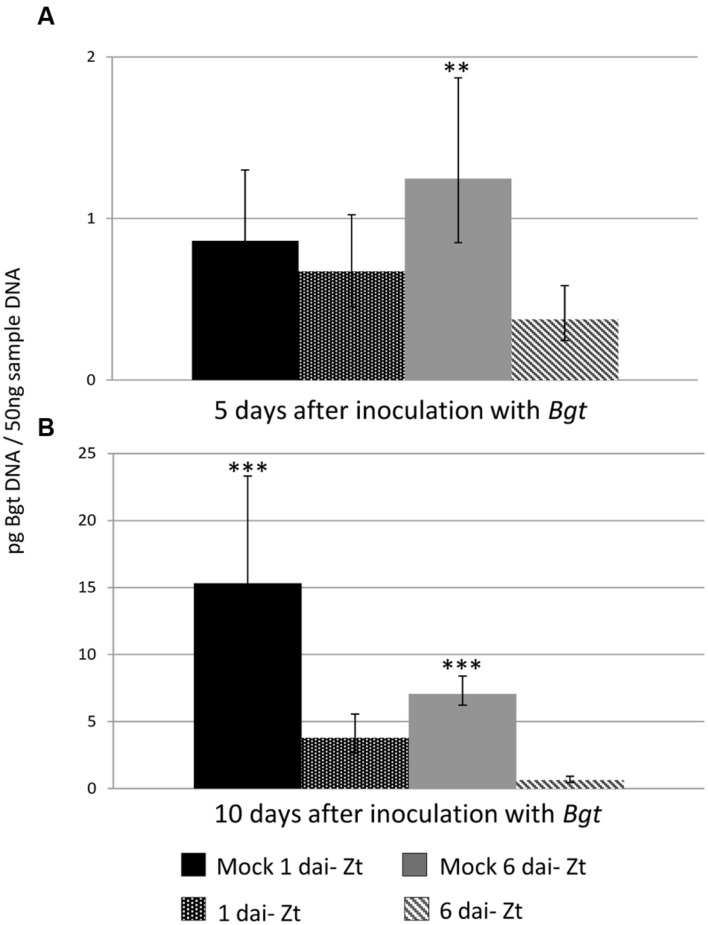
**Biomass of *B. graminis* f.sp. *tritici* (*Bgt*) on wheat leaves, measured as *Bgt* DNA as a proportion of total DNA.** Leaves of Longbow were pre-inoculated with virulent *Z. tritici* IPO323 or mock inoculated either 1 or 6 days before inoculation with virulent *Bgt* isolate JIW48. Samples were taken at 5 **(A)** and 10 days **(B)** after mildew inoculation. *Bgt* DNA (pg per 50 ng sample DNA) was determined by RT-qPCR using a Taqman probe. ^∗∗^*P* < 0.1, ^∗∗∗^*P* < 0.001 based on least significant differences of predicted means.

RT-qPCR was used to determine the concentration of *Bgt* DNA on infected leaves as a proportion of total DNA. The timing of *Z. tritici* inoculation (1 or 6 days), the treatment (*Z. tritici* or mock) and the number of days after *Bgt* inoculation that the samples were taken (5 or 10) all had a significant effect on the level of fungal biomass in the leaves (**Table 1**). The amount of *Bgt* DNA was greater in all samples collected 10 days after inoculation than in those collected after 5 days (*P* < 0.001). In samples inoculated with *Bgt* 6 dai with *Z. tritici*, there was less *Bgt* than in those inoculated with *Bgt* 1 dai with *Z. tritici* (*P* < 0.01). In the 10 days samples, there was significantly less *Bgt* in samples inoculated with *Bgt* at both 1 and 6 dai with *Z. tritici* than in the mock samples (*P* < 0.001). At 5 days this difference was only apparent in the samples inoculated with *Bgt* at 6 dai with *Z. tritici* (*P* < 0.01; **Figure 7**).

**Table 1:** Accumulated analysis of variance table from logistic regression model: interval^∗^daiBg^∗^trt where the ^∗^ operator indicates that both the main effects and the interaction of the factors were estimated.

Change	d.f.	m.s.	v.r.	F pr
Interval	1	1.3589	8.85	0.007
daiBg	1	2.9516	19.23	<0.001
trt	1	1.8015	11.74	0.002
Interval.daiBg	1	1.1335	7.38	0.012
Interval.trt	1	0.0756	0.49	0.49
daiBg.trt	1	0.278	1.81	0.191
Interval.daiBg.trt	1	0.0679	0.44	0.512
Residual	24	0.1535		
				
Total	31	0.3662		


*Interval indicates the amount of time after inoculation with *Z. tritici* that *Bgt* inoculation occurred (either 1 or 6 days). DaiBg is the time that the samples were taken, either 5 or 10 days after *Bgt* inoculation. Trt is the treatment of *Z. tritici* or mock inoculation.*

## Discussion

We investigated the three-way interaction between wheat and two economically important, specialist fungal pathogens: an obligate biotroph, *B. graminis* f.sp. *tritici* and a necrotroph, *Z. tritici*. The experiments established a laboratory system to allow these two pathogens to infect the same host plant simultaneously. The main findings are that a compatible interaction between *Z. tritici* and the wheat leaf reduces the number, size, and reproductive capacity of mildew colonies that a normally virulent *Bgt* isolate produces but does not significantly alter the early development of *Bgt* on the leaf. Conversely, an incompatible interaction between *Z. tritici* and the wheat leaf has no apparent effect on the ability of a virulent *Bgt* isolate to form mildew colonies and does not detectably alter the susceptibility of the leaf to an avirulent *Bgt* isolate. The effect on virulent *Bgt* was not elicited by non-viable spores of *Z. tritici*. Increased resistance to *Bgt* is therefore elicited specifically by infection with virulent *Z. tritici*. This occurs before Septoria symptoms are visible on the leaf, implying that the effect involves a physiological interaction during the latent, endophytic period of *Z. tritici*, which either takes place directly between this fungus and *Bgt* or is mediated by the wheat leaf.

Priming of plant defenses which are induced in response to one pathogen and are effective against future attack by another pathogen has been suggested as a mechanism which inhibits subsequent pathogen growth (e.g., Lyngs Jørgensen et al., 1998; Lyngkjær and Carver, 2000; Aghnoum and Niks, 2012). Here, *Bgt* spores were able to infect and develop appressoria, haustoria, and ESH but were hindered in subsequent growth and reproductive ability at the later stages of fungal development. This implies that the suppressive effect of *Z. tritici* is not effective against the early stages of development of *Bgt*. The inhibition of *Bgt* by *Z. tritici* was consistent regardless of the length of the interval between the inoculations with the two fungi, which implies that the effect is unlikely to be a specific effect of the later developmental stages of *Z. tritici*. In particular, it is presumably an effect of the early, endophytic phase of the *Z. tritici* life cycle, not the later, necrotrophic phase, because *Bgt* was inhibited before Septoria symptoms were visible. The lack of a detectable effect of non-viable spores of *Z. tritici* on mildew colony formation could mean that MAMPs delivered to the surface of the wheat leaf are not sufficient to inhibit growth and development of *Bgt*. The method is not sensitive enough to determine if proteinaceous MAMPs are involved as these may be destroyed by the autoclaving process. Additionally, during the period of symptomless colonization there is no apparent nutrient acquisition from the host (Rudd et al., 2015), which means that *Bgt* is unlikely to be hindered in obtaining nutrients from the plant.

An explanation for the inhibition of *Bgt* through pre-infection of wheat by *Z. tritici* may concern the very different roles of host responses to the two diseases. In powdery mildew, the hypersensitive response, involving death of infected epidermal cells and subtending mesophyll cells, plays a critical role in inhibition of avirulent *B. graminis* (Boyd et al., 1995) whereas *Bgt*, as an obligate biotroph, requires living host tissue for its growth, development, and reproduction. In Septoria, by contrast, fungal development in a compatible interaction occurs in necrotic tissue but in an incompatible interaction, the host plant keeps its leaf tissue alive, possibly by suppressing the hypersensitive response and thus preventing transition of *Z. tritici* to the necrotrophic phase (Keon et al., 2007; Jing et al., 2008). It has been proposed that *Z. tritici* hijacks disease resistance signaling pathways (Hammond-Kosack and Rudd, 2008), causing its host to express a response which is typical of resistance to biotrophic pathogens but elicits susceptibility to the necrotrophic *Z. tritici*. We propose that as yet unknown early signaling events during the latent phase of the interaction between wheat and the virulent genotype of *Z. tritici*, prior to the necrotic stage, can lead to the suppression of the obligate biotroph *Bgt*.

Higher inoculum levels of *Z. tritici* reduced the number of mildew colonies formed on the leaf in a dosage dependent manner. The severity of Septoria symptoms is itself closely correlated with inoculum load (Fones et al., 2015), which is consistent with the response of wheat to *Z. tritici* being localized rather than systemic within the leaf (Rudd et al., 2008). At higher doses of *Z. tritici*, therefore, it is expected that a larger proportion of the leaf is involved in the proposed early signaling events. The greater the number of *Z. tritici* infection sites, the larger the impact on the mildew colonies will be as *Bgt* spores are dispersed over the entire leaf. This is consistent with the hypothesis that infection by *Z. tritici* may predispose leaf tissue around the site of infection to become necrotic and thus to have reduced susceptibility to *Bgt*.

By contrast, the incompatible *Z. tritici* isolate had no effect on the outcome of *Bgt* infection. This may be because early, endophytic infection by avirulent *Z. tritici* does not yet induce the plant’s mechanism which suppresses the necrotising response to *Z. tritici* infection (Hammond-Kosack and Rudd, 2008) or because any such host response does not further increase the susceptibility of wheat leaf tissue to *Bgt*.

The ability of *Bgt* to form appressoria and haustoria and to produce secondary hyphae despite the inhibition of later development by pre-inoculation with virulent *Z. tritici* contrasts with the interaction between *Z. tritici* and *P. striiformis*. In the latter case, germination of urediniospores was reduced by the presence of *Z. tritici* and rust development was restricted to areas of the leaf not infected by *Z. tritici* (Madariaga and Scharen, 1986), suggesting the rust pathogen could not compete for resources in the face of *Z. tritici* infection.

Longer incubation periods of the detached leaves prior to inoculation with *Bgt* has a significant negative impact on *Bgt* infection, as is apparent from both colony number and fungal biomass quantification. It is likely that this is due to deterioration of the leaf tissue once it is detached from the plant. All the experiments presented here included appropriate mock-inoculated controls which also showed a reduction in colony formation in each treatment. Detached leaves were used because they allow many treatment combinations to be tested in replicate in controlled conditions (Arraiano et al., 2001).

The results presented here indicate that because susceptibility to *Z. tritici* inhibits powdery mildew, breeding efforts should focus on increasing resistance to Septoria whilst maintaining the current level of moderately high partial resistance to powdery mildew in many winter wheat breeding programs (Brown, 2015). Resistance to *Z. tritici* had no effect on *Bgt*, so focusing attention on breeding for Septoria resistance should not have a detrimental effect on mildew resistance. In the U.K., breeding for mildew resistance has taken place for many years, whereas breeding for resistance to Septoria has been relatively recent but is now one of the main targets for new winter wheat varieties (Brown, 2015). Breeders and farmers require acceptable resistance to all diseases so more information on how different pathogens interact with each other is desirable when breeding new varieties, especially in the face of growing government and public concern over fungicides (Torriani et al., 2015). There is little value in having good resistance to one disease if its resistance to another is poor as this will not reduce demand for pesticides. Speculatively, long term breeding for resistance to powdery mildew may have meant that wheat is now well adapted to defense against biotrophic pathogens, but less well adapted to defense against necrotrophic pathogens.

We have shown that a non-biotrophic pathogen has a negative impact on a biotrophic pathogen. Histology of powdery mildew development shows that *Z. tritici* infection inhibits growth and reproductive capacity of *Bgt*, the powdery mildew fungus. This effect is apparent before necrotic symptoms indicative of Septoria disease are visible. Understanding the interactions of host responses to diverse pathogens with differing life-histories will underpin efforts to breed crop varieties with durable resistance to several diseases that occur simultaneously.

## Author Contributions

EO and JB planned the research, analyzed the data, and wrote the paper. ESO did the experiments.

## Conflict of Interest Statement

The authors declare that the research was conducted in the absence of any commercial or financial relationships that could be construed as a potential conflict of interest.

## Footnotes

1 http://www.ebi.ac.uk/goldman-srv/webprank/

2 http://www.ncbi.nlm.nih.gov
