# Supplementary material for: Reduction of Growth and Reproduction of the Biotrophic Fungus Blumeria graminis in the Presence of a Necrotrophic Pathogen
Source: Front Plant Sci. 2016 May 31;7:742. doi: 10.3389/fpls.2016.00742 (PMC4885842; doi:10.3389/fpls.2016.00742)
Supplement: Supplementary file 1 [file Table_1.DOCX]

Table S1. Accumulated analysis of deviance at 8, 24/32 and 48 hours after inoculation with *Blumeria graminis f. sp. tritici* from logistic regression model Rep+Day*Trt where the * operator indicates that both the main effects and the interaction of the factors were estimated. Day indicates the amount of time after inoculation with Z*ymoseptoria tritici* that *Bgt* inoculation occurred (either 1 or 6 days) and Trt indicates the treatment of *Z. tritici* inoculation or mock-inoculation on either Flame or Longbow.

Accumulated analysis of deviance 8 hai

mean deviance

Factor d.f. deviance deviance ratio F pr.

Rep 1 2.650 2.650 0.74 0.395

Day 1 0.624 0.624 0.17 0.678

Trt 3 3.382 1.127 0.32 0.814

Day.Trt 3 20.224 6.741 1.88 0.148

Residual 39 139.485 3.577

Accumulated analysis of deviance 24/32 hai

mean deviance

Factor d.f. deviance deviance ratio F pr.

Rep 1 18.027 18.027 5.71 0.022

Day 1 1.135 1.135 0.36 0.552

Trt 3 10.855 3.618 1.15 0.343

Day.Trt 3 3.241 1.080 0.34 0.795

Residual 39 123.177 3.158

Accumulated analysis of deviance 48 hai

mean deviance Factor d.f. deviance deviance ratio F pr.

Rep 1 1.018 1.018 0.38 0.540

Day 1 56.794 56.794 21.35 <.001

Trt 3 14.506 4.835 1.82 0.160

Day.Trt 3 2.015 0.672 0.25 0.859

Residual 39 103.729 2.660
